# Genome-Wide Identification of QTLs for Grain Protein Content Based on Genotyping-by-Resequencing and Verification of *qGPC1-1* in Rice

**DOI:** 10.3390/ijms21020408

**Published:** 2020-01-09

**Authors:** Yi-Bo Wu, Guan Li, Yu-Jun Zhu, Yi-Chen Cheng, Jin-Yu Yang, Hui-Zhe Chen, Xian-Jun Song, Jie-Zheng Ying

**Affiliations:** 1 State Key Laboratory of Rice Biology and Chinese National Center of Rice Improvement, China National Rice Research Institute, Hangzhou 310006, China; 15978677129@163.com (Y.-B.W.); li@ricescience.org (G.L.); yjzhu2013@163.com (Y.-J.Z.); 13872209530@163.com (Y.-C.C.); yang_15271211512@163.com (J.-Y.Y.); chenhuizhe@163.com (H.-Z.C.); 2 Key Laboratory of Plant Molecular Physiology, Institute of Botany, the Chinese Academy of Sciences, Beijing 100093, China; songxj@ibcas.ac.cn

**Keywords:** quantitative trait locus, grain protein content, single nucleotide polymorphism, residual heterozygote, rice (*Oryza sativa*), specific length amplified fragment sequencing, Kjeldahl nitrogen determination, near infrared reflectance spectroscopy

## Abstract

To clarify the genetic mechanism underlying grain protein content (GPC) and to improve rice grain qualities, the mapping and cloning of quantitative trait loci (QTLs) controlling the natural variation of GPC are very important. Based on genotyping-by-resequencing, a total of 14 QTLs were detected with the Huanghuazhan/Jizi1560 (HHZ/JZ1560) recombinant inbred line (RIL) population in 2016 and 2017. Seven of the fourteen QTLs were repeatedly identified across two years. Using three residual heterozygote-derived populations, a stably inherited QTL named as *qGPC1-1* was validated and delimited to a ~862 kb marker interval JD1006–JD1075 on the short arm of chromosome 1. Comparing the GPC values of the RIL population determined by near infrared reflectance spectroscopy (NIRS) and Kjeldahl nitrogen determination (KND) methods, high correlation coefficients (0.966 and 0.983) were observed in 2016 and 2017. Furthermore, 12 of the 14 QTLs were identically identified with the GPC measured by the two methods. These results indicated that instead of the traditional KND method, the rapid and easy-to-operate NIRS was suitable for analyzing a massive number of samples in mapping and cloning QTLs for GPC. Using the gel-based low-density map consisted of 208 simple sequence repeat (SSR) and insert/deletion (InDel) markers, the same number of QTLs (fourteen) were identified in the same HHZ/JZ1560 RIL population, and three QTLs were repeatedly detected across two years. More stably expressed QTLs were identified based on the genome resequencing, which might be attributed to the high-density map, increasing the detection power of minor QTLs. Our results are helpful in dissecting the genetic basis of GPC and improving rice grain qualities through molecular assisted selection.

## 1. Introduction

Rice grain quality, including appearance, milling, cooking and eating, as well as nutritional qualities, determines the market value, and is getting more and more concern from rice researchers, producers and consumers [1,2]. Grain protein content (GPC) is not only one key factor determining nutritional quality, but is also closely associated with cooking and eating qualities [3,4]. Generally, the increase of GPC may consequently lead to low eating quality. 

Compared to the protein content of other cereal crops such as wheat and barley, GPC in rice is relatively low, with a mean about 8.0% and a range of 4.9% to 19.3% in the *indica* subspecies and 5.9% to 16.5% in the *japonica* subspecies [5]. As a typical quantitative trait, GPC in rice is easily affected by environmental conditions, especially the level of nitrogen fertilizer in the field, which makes it very difficult to manipulate in a traditional breeding program. Therefore, illuminating the genetic basis of GPC makes a lot of sense in constructing a molecular marker-assisted selection system to improve rice grain quality [6,7].

Quantitative trait locus (QTL) analysis is the main strategy for dissecting the genetic mechanism underlying a target quantitative trait. During the past two decades, hundreds of QTLs for GPC in rice were detected throughout the entire 12 chromosomes, using different mapping populations, including the recombinant inbred line (RIL) [3,8,9,10,11,12,13], double haploid population [14,15,16,17], chromosome segment substitution line [4,18,19] and the backcross-inbred population [20]. As GPC is sensible to environmental factors, QTLs controlling the GPC are difficult to be repeatedly identified in different populations, or in the same population under different environments [19]. Till now, only two QTLs, *qPC1* and *qGPC-10*, have been map-based cloned and functionally analyzed. *qPC1* was found on the long arm of chromosome 1, which encodes a putative amino acid transporter OsAAP6 and functions as a positive regulator of GPC in rice [6]. *qGPC-10* located on chromosome 10 encodes a glutelin type-A2 precursor, and is also a positive regulator of GPC [7]. Besides, another stably inherited QTL *qPC-1* that is nonallelic to *qPC1* was validated and delimited to a 41-kb region on the long arm of chromosome 1 [19]. Owing to the detection instability of GPC QTLs, it is important to confirm the genetic effect of the QTLs detected in the primary mapping before their map-based cloning and application in the improvement of rice nutritional quality.

Genotyping and phenotyping of the mapping population are two essential components for QTL analysis. With the development of next-generation sequencing, genotyping-by-sequencing becomes a feasible technique to rapidly identify a huge number of single nucleotide polymorphisms (SNPs) throughout the whole genome. Then, a high-density linkage map can be constructed with saturated SNP markers, while most current maps are low-density, and only contain hundreds of gel-based DNA markers, such as restriction fragment length polymorphism and simple sequence repeat (SSR) markers. The detection power of minor QTLs can be improved, and the confidence interval of the QTL can be reduced in a high-resolution linkage map [21]. Among the most common genotyping-by-sequencing methods, specific length amplified fragment sequencing (SLAF-seq) is acceptable as an efficient and high-resolution technology with a relatively lower sequencing cost [22]. With the availability of rice genome draft, genotyping-by-resequencing (GBR) has been applied in linkage mapping and genome-associated analysis to map QTLs for important agronomic traits. Nevertheless, for GPC, the information of QTL identified by GBR of a bi-parent population is still limited in rice.

GPC is traditionally measured by the Kjeldahl nitrogen determination (KND), which is time-consuming and needs a large amount of chemicals such as strong acid and alkali. Therefore, this KND method is difficult for the measurement of a massive number of samples, which is usually necessary in the map-based cloning of a target QTL. Compared to the KND method, near infrared reflectance spectroscopy (NIRS) is a promising technique that is fast and easy-to-use [23]. A lot of QTLs were identified, and two major QTLs have been successfully isolated through the NIRS method. However, it keeps unknown whether there is difference in the detection power of QTLs for GPC between the two methods.

In this study, we mainly completed the following research objectives. First, we analyzed the correlation between the GPC values measured by the KND and NIRS methods, and validated the feasibility of mapping QTLs for GPC using NIRS instead of KND. Second, we identified QTLs for GPC with a high-resolution genetic map containing 18,194 SNP markers in an RIL population, which was derived from a cross between an *indica* variety Huanghuazhan (HHZ) and a *japonica* accession Jizi1560 (JZ1560). Third, we compared the GPC QTLs identified with the high- and low-density map in the same HHZ/JZ1560 RIL population. Finally, one stably inherited major QTL (*qGPC1-1*) located on the short arm of chromosome 1 was validated using three secondary populations developed from three residual heterozygotes (RH) with the heterozygous genotype at the target interval.

## 2. Results

### 2.1. Phenotypic Variation and Correlation Analysis

**Figure 1:**
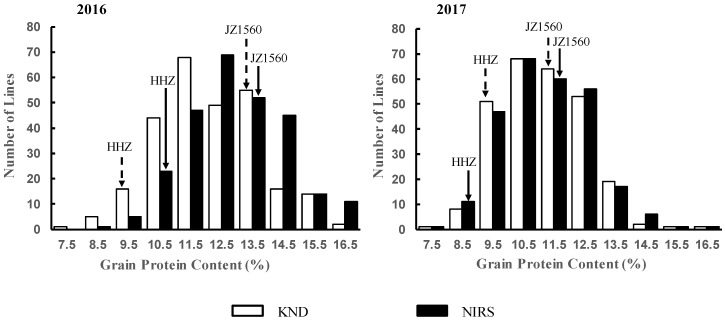
Frequency distributions of grain protein content (GPC) measured by near infrared reflectance spectroscopy (NIRS) and Kjeldahl nitrogen determination (KND) methods in the recombinant inbred line (RIL) population. Parental values are indicated by arrows.

In 2016 and 2017, the GPC of the HHZ/JZ1560 RIL population and the parents was measured by NIRS and KND, respectively. The descriptive statistics of phenotypic variation are shown in Table 1. Significant phenotypic differences were observed between the parents, and the GPC of JZ1560 was higher than that of HHZ in both years. Frequency distributions of GPC in the brown rice flour of RILs and the parents were plotted (Figure 1). The GPC of the RILs showed similar distribution either measured by the KND or NIRS method in each year. Phenotypic variation was continuously distributed with low skewness and kurtosis, showing a typical pattern of quantitative variation. Using the KND method, the GPC of the RILs showed a wide variation from 7.27% to 15.78% in 2016 and from 7.39% to 15.98% in 2017 (Figure 1). Continuous segregation and significant transgressive segregation at two directions suggested polygenic control underlying this trait.

Significant positive correlations between the GPC values measured by the two methods or in different years were found in the RIL population (Table 2). However, correlation coefficients for GPC determined by KND and NIRS methods as 0.966 in 2016 and 0.983 in 2017 were much higher than that of GPC measured with the same method in different years as 0.638 for NIRS and 0.631 for KND. This implied that GPC determined by NIRS was quite consistent with that by the KND method, and GPC was strongly influenced by environmental factors.

### 2.2. QTL Analysis of Grain Protein Content in the HHZ/JZ1560 RIL Population

**Figure 2:**
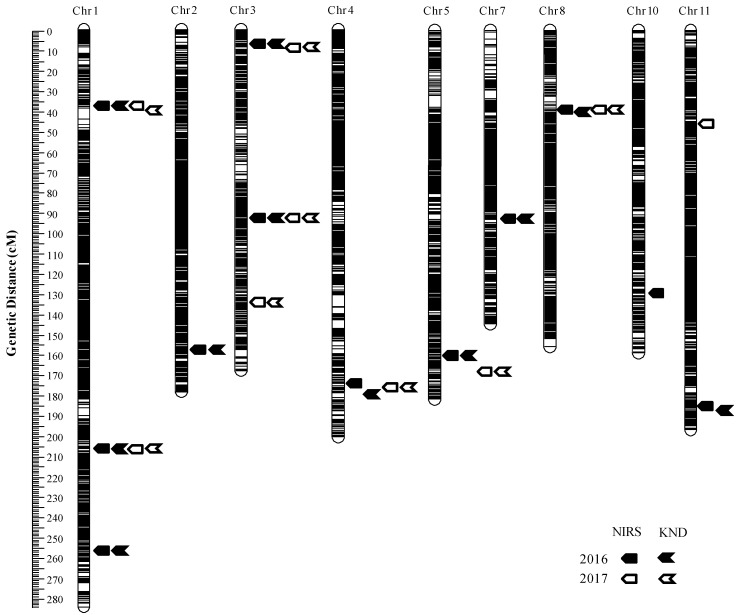
High-density linkage map based on genotyping-by-resequencing showing the most likely positions of QTLs for GPC measured by NIRS and KND methods in the HHZ/JZ1560 RIL population.

**Figure 3:**
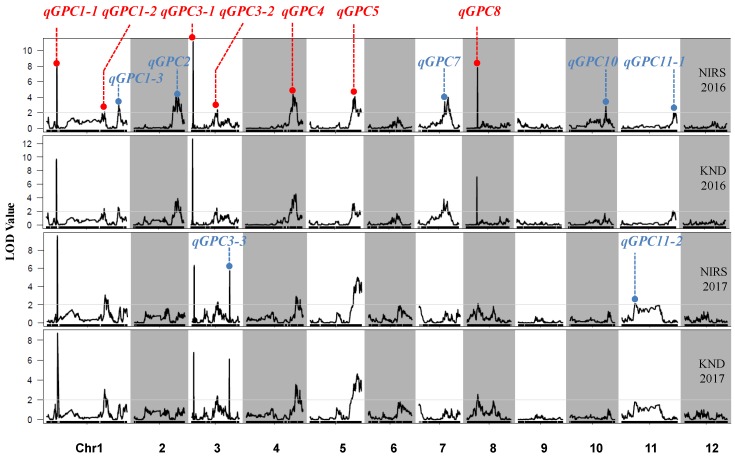
The identified QTLs for GPC measured by the NIRS and KND methods through analyzing the SNP genotypes and corresponding phenotypes of the 280 RILs. Red font indicates that QTLs were detected in both 2016 and 2017, and blue font indicates that QTLs were identified in either year 2016 or 2017.

Combining the high-density genetic map containing 18,194 SNP markers with the GPC means of each RIL, 14 QTLs were detected on the whole genome except for chromosomes 6, 9 and 12 with each QTL explaining 0.81%–18.59% of the phenotypic variations (Table 3, Figure 2 and Figure 3). Among the QTLs, 12 were identified in 2016 and nine in 2017. The detailed description of each QTL including peak location, peak LOD value, additive effect and percentage of total phenotypic variations (*R*^2^) are showed in Table 3. Except for *qGPC2*, *qGPC8* and *qGPC10*, the enhancing alleles for GPC were derived from JZ1560 at the remaining 11 loci as the brown rice of JZ1560 contained significantly higher GPC (Table 1).

In order to find the difference in the detection power of QTLs using different measurement methods of GPC, we further compared the QTLs for GPC determined by the NIRS and KND methods. In 2016, we identified 12 QTLs for GPC measured by NIRS and 11 QTLs for GPC measured by KND, which explained 55.07% and 48.25% of the total phenotypic variations, respectively. Eleven QTLs were commonly mapped using the two measurement methods, and one QTL (*qGPC10*) with a small genetic effect was only detected using the NIRS method (Figure 2 and Figure 3, Table 3). Similar results were observed in 2017. Eight common QTLs were identified by both the measurement methods, and only one minor QTL (*qGPC11*) was mapped on chromosome 11 using the NIRS method. This indicated that QTLs for GPC were coincided between the two GPC measurement methods. Seven of the fourteen QTLs, *qGPC1-1*, *qGPC1-2*, *qGPC3-1*, *qGPC3-2*, *qGPC4*, *qGPC5* and *qGPC8*, were repeatedly identified in both years. The remaining seven QTLs were only detected in one year.

**Figure 4:**
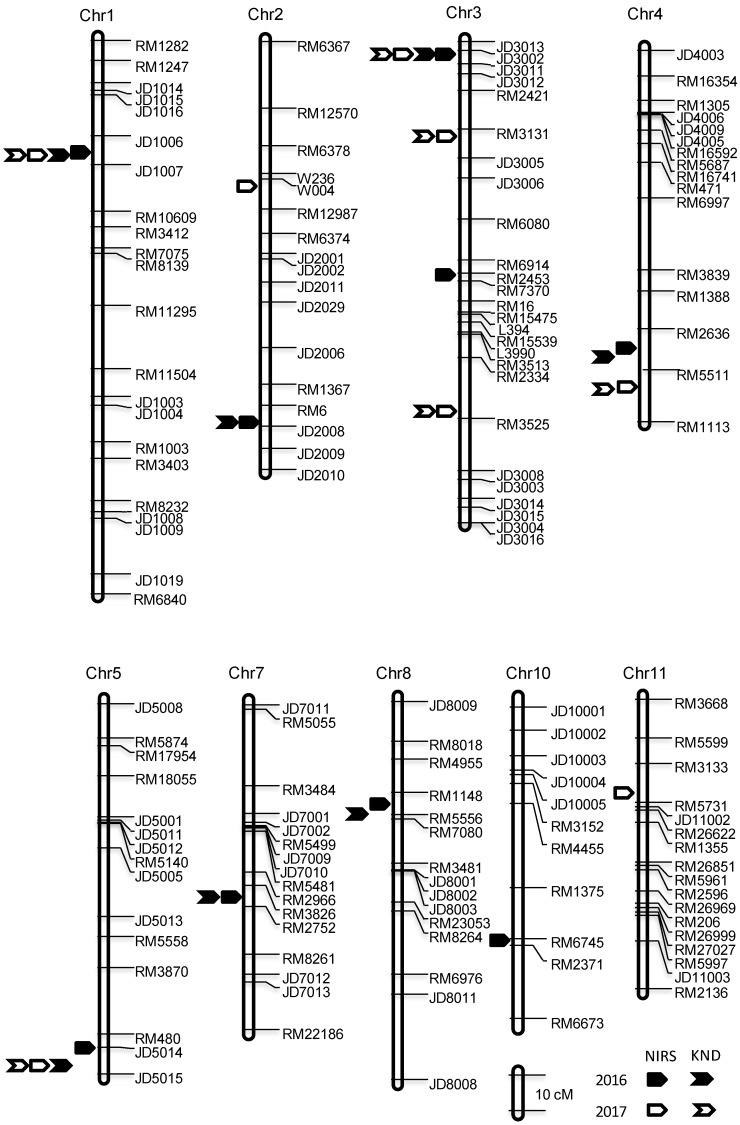
Low-density linkage map containing 208 gel-based SSR and InDel markers showing the most likely positions of QTLs for GPC measured by NIRS and KND methods in the HHZ/JZ1560 RIL population.

QTL analysis was also performed using a low-density genetic map containing 208 SSR and InDel markers and a total of 14 QTLs were detected in the same HHZ/JZ1560 RIL population (Figure 4, Table S1). Compared with the seven stably inherited QTLs identified in the high-density genetic map, only three QTLs including *qGPC1*, *qGPC3-1* and *qGPC5* were mapped at the same region and showed the similar effects for the two years using the low-density genetic map, suggesting that the high-density genetic map increased the detection power of QTLs for GPC.

The *qGPC1-1* was detected on the short arm of chromosome 1 across two years using the high-density genetic map and accounted for 9.14% to 11.85% of the phenotypic variations. The allele from JZ1560 at this locus increased GPC by 0.47%-0.58%. Corresponding to *qGPC1-1*, *qGPC1* was mapped at the same location with the flanking markers JD1006 and JD1007 using the low-density genetic map in both years (Figure 4, Table S1). The *qGPC1* identified in this study contributed 11.78% to 13.33% of phenotypic variations with a relatively large additive effect ranging from 0.54% to 0.71% (Table S1). 

### 2.3. Validation and Delimitation of qGPC1-1 Using RH-derived F_2_ Populations

To confirm the genetic effect and location of *qGPC1-1*, three RH individuals were selected from one F_8_ RIL line with the heterozygous genotype covering the target marker interval of JD1006–JD1007. Three RH-derived F_2_ populations, named as WB01, WB02 and WB03, were developed from the three plants with sequential heterozygous segments extending from JD1006 to JD1007, respectively. Based on the sequence differences between the parents HHZ and JZ1560 identified by 30-fold whole genome re-sequencing, additional six InDel markers were developed and used to genotype the three populations (Table S2). GPC was continuously distributed and ranged from 8.33% to 11.90%, 8.27% to 12.19% and 8.32% to 10.47% in WB01, WB02 and WB03 populations, respectively (Figure S1). 

**Figure 5:**
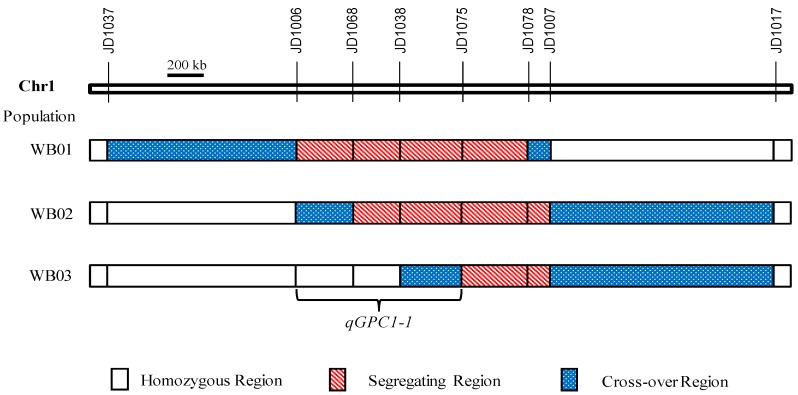
Genotypic compositions of the three residual heterozygote-derived F_2_ populations in the segregating regions.

Three segmental linkage maps were constructed for WB01, WB02 and WB03, respectively (Figure 5). Combined the genotype and phenotype information, *qGPC1-1* was identified in WB01 and WB02 populations, with the JZ1560 allele always increasing GPC (Table 4; Figure 5). This QTL explained 26.00% and 27.40% of phenotypic variations with the similar additive effects of 0.36% and 0.39% in WB01 and WB02, respectively. No QTL for the GPC was detected in the WB03 population. Therefore, *qGPC1-1* should be located within the common segregating regions of WB01 and WB02, but outside the segregating region of WB03. As shown in Figure 5, *qGPC1-1* was delimited to the interval between JD1006 and JD1075 (~862 kb) with a common segregating region JD1068–JD1075 and one flanking cross-over region JD1006–JD1068.

## 3. Discussion

Elucidating the genetic mechanism of GPC accumulation is very important for regulating rice grain qualities in breeding. In the present study, we characterized the genetic basis of GPC and identified a total of 14 QTLs using the high-resolution map in the HHZ/JZ1560 RIL population. Although GPC is sensitive to environmental conditions and the QTLs for GPC are difficult to be repeatedly identified in different environments, the majority of the 14 QTLs have been reported in the previous studies. On the long arm of chromosome 1, QTLs for GPC have been reported in some studies including *pro1* between RM226 and RM297 [15], *qPC-1* between R888 and R1485 [13], *qPC1* between RM472 and RM104 [6], *qPC-1* between RM1196 and RM302 [19] and *TGP1b* between RM1297 and RM1067 [4]. The *qGPC1-2* and *qGPC1-3* were located in the adjacent chromosome regions with these reported QTLs, and the *indica* variety HHZ allele decreased the GPC. Two minor QTLs, *qGPC2* and *qGPC11-1*, were also mapped at the similar locations with *pro2* between RM6 and RM112 and *pro11* between RM209 and RM229 [15]. On the chromosome 3, we detected three QTLs including *qGPC3-1*, *qGPC3-2* and *qGPC3-3*, and the enhancing alleles were all from the *japonica* rice JZ1560. The *qGPC3-1* with the largest effect in 2016 and *qGPC3-2* were repeatedly detected as *qPC-3.1* in the interval XNpb212–G1318 and *qPC-3.2* in the interval R758–XNpb15, respectively [13]. The *qGPC3-3* was located in the overlapping confidence interval with the QTLs for protein content in several previous reports [13,14,18]. These results indicated that there are multiple genetic factors controlling GPC on chromosome 3. The *qGPC4* was detected within the *qPC-4* region between RG214 and RG620 [12]. We still noted that *qGPC5* was repeatedly identified as *qRPC5* for rice protein content in the interval RG435–RG172a using a doubled haploid population [16]. Although *qGPC7* was only detected in 2016, four QTLs were located in the same or adjacent regions as reported by previous studies [3,4,8,17]. On the chromosome 8, *qGPC8* showed overlapping intervals with *cp8.1*, *qPC-8a* and *TGP8*, which have been identified using different populations in different environments [4,18,24]. The *qPC11-2* with minor effect was mapped near to *qPC11* between RM202 and RM206 [25]. No QTL for GPC has been mapped in the region of *qGPC10* on chromosome 10 before, therefore *qGPC10* might be newly detected in this study. Unlike many previous studies, we did not detect the QTL for GPC near to the *Wx* locus on chromosome 6 [4,8,12,25]. Over all, the locations of QTLs for GPC showed a significant similarity between our studies and the previous findings.

Of the 14 QTLs identified in this study, the *qGPC1-1* was a stably expressed QTL with a relatively large effect and it was repeatedly detected in both years. This QTL was also identified in the similar chromosome region in different populations and environments, implying that *qGPC1-1* plays an important role in controlling GPC [4,11,18]. Based on the primary mapping result, *qGPC1-1* was further validated and delimited in the interval JD1006–JD1075, corresponding to the 6.0–6.8 Mb region on the short arm of chromosome 1 in the Nipponbare genome [26]. The *japonica* rice JZ1560 allele contributed to the increase of GPC in the RH-derived F_2_ population (Table 4). Dissecting the genetic mechanism underlying GPC is important for the improvement of rice grain quality, and the main obstacle to date is the absence of key genes/QTLs regulating GPC. Primary mapping leads to a large confident interval and poor repeatability of target QTLs, which makes it difficult to find tightly linked markers for marker-assisted selection. Validation and delimitation of *qGPC1-1* contributed to the facilitation of marker-assisted selection in rice breeding for high nutritional quality. Furthermore, based on these results, fine mapping and map-based cloning of *qGPC1-1* is under way.

Mapping and isolation of QTLs need a high efficiency method to measure GPC. Cloning of QTLs controlling the natural variation of GPC is the important step toward uncovering the regulatory mechanism underlying this quantitative trait. However, map-based cloning of a QTL needs phenotype and genotype information of a massive number of samples, suggesting a rapid and easy operation method for GPC is necessary. Using the NIRS system, the GPC value can be directly measured once the brown rice is grinded into flour. 

Compared with the NIRS, the KND method for GPC in rice needs further lengthy operation, which is time-consuming and laborious. More importantly, high correlation between the GPC values determined by the NIRS and KND methods was observed in the HHZ/JZ1560 RIL population across two years. Only two minor QTLs (*qGPC10* and *qGPC11-1*) were detected using the single measurement method of GPC, the remaining twelve QTLs were identical to be identified by both methods in 2016 or 2017 (Figure 2 and Figure 3, Table 3). Comparative analysis between the two methods suggested that NIRS could be a feasible strategy for the mapping and map-based cloning of QTLs for GPC instead of the KND method. In recent years, NIRS has been successfully employed in the isolation and characterization of two major QTLs for GPC, *qPC1* and *qGPC-10* [6,7].

Accompanied with the development of DNA sequence techniques, the sequencing cost decreases continuously and more and more high-density genetic maps have been constructed to detect QTLs for different traits through genotyping-by-sequencing [21,27,28]. In the present study, QTLs for GPC were mapped simultaneously using a high-density genetic map with 18,194 SNP markers identified by GBR and a low-density map with 208 gel-based SSR and InDel markers in the same RIL population. Although the same number of QTLs were identified using the different resolution genetic maps, 7 of 14 and 3 of 14 QTLs were repeatedly detected across two years in the high- and low-density genetic maps, respectively. Identification of more stably expressed QTLs might be attributed to the increased detection power resulted by the saturated SNP markers in the high-density genetic map.

## 4. Materials and Methods

### 4.1. Plant Materials and Field Experiments

The HHZ/JZ1560 mapping population consisting of 280 RILs was developed using the single-seed descendent method by Ying et al. [29]. HHZ is an *indica* variety as the female parent with small grains and being widely cultivated in China, while JZ1560 is a *japonica* rice accession with very large grains (Figure S2). Three RH-derived F_2_ populations (WB01, WB02 and WB03) consisting 180, 137 and 115 individuals, respectively, were originated from 3 RH plants that were selected from one line of the F_7_ HHZ/JZ1560 RIL population. The three populations were used to validate the target QTL, *qGPC1-1*.

All the populations, together with the two parents, were planted with the spacing of 16.7 cm between plants and 26.7 cm between rows during the rice-growing seasons in the experimental field of China National Rice Research Institute, Hangzhou (120.2° E, 30.3° N), China. Eighteen plants per RIL were transplanted and the middle four plants were harvested in bulk at maturity for the measurement of GPC in 2016 and 2017. The three RH-derived populations were tested for one year in 2018. Field management was conducted according to the common practice in rice production. Fertilizer was applied for each cultivation year as follows: 375 kg/ha compound fertilizer (N-P_2_O_5_-K_2_O: 14:16:15) as the basal fertilizer, 52 kg/ha nitrogen at seedling stage, 69 kg/ha nitrogen at 5 days after transplanting and 150 kg/ha potassium at 20 days after transplanting.

### 4.2. GPC Measurement

GPC of brown rice flour for each RIL was carefully measured by the KND and NIRS methods, respectively. Rice grains were harvested after maturity and stored at room temperature for at least three months before the GPC measurement. The fully filled grains were de-hulled into brown rice by a rice huller (JLGJ-45, Taizhou, Zhejiang, China), then the brown rice was grinded into flour by a grinder (IKA TUBE-MILL, Staufen, Germany). Brown rice flour samples were directly used to determine GPC by NIRS (Foss, Sweden). Each sample was assayed twice and the mean values were used for further analysis. GPC was calculated according to the modified NIRS model constructed by Xie et al. [23].

For the KND method to measure GPC, 0.2 g brown rice flour, 1.0 g catalyst (CuSO_4_:Na_2_SO_4_ = 1:10) and 4.0 mL of H_2_SO_4_ were first added into a 100-mL digestion tube in turn. Then the mixture was immediately heated at 420 °C for 2 h in a digestion stove. After the solution being digested, the mixture was cooled to room temperature. Then, 10 mL ddH_2_O was added into the digestion tube. The mixed solution was analyzed using a Kjeltec 8400 Autoanalyzer (Foss, Sweden). GPC of the rice flour was calculated from the total N content by multiplying a conversion factor of 5.95 [30]. The assay for each sample was conducted with two replicates and the means were used for further analysis.

### 4.3. Genetic Map and DNA Marker Analysis

To compare the detection power of GPC QTL, high-density and low-density linkage maps of the RIL population were both used to map QTL, respectively. The high-density linkage map constructed by one of the GBR method, specific length amplified fragment, was composed of 18,194 SNP markers and spanned 2132.56 cM with an average genetic distance of 0.12 cM. In our previous study, we constructed the SLAF library for each RIL and the products were further sequenced using Illumina HiSeq 2500 system (Illumina, Inc.; San Diego, CA, USA) [29]. Polymorphism loci between the parents were identified for the selection of high-quality SNP markers after filtering out the low-quality raw reads. SNP markers with more than 20% missing data and the segregation distortion were further filtered out. A total of 18,194 high quality SNP markers were used to genotype the 280 RILs. The low-density linkage map constructed by the gel-based method consisted of 121 SSR and 87 InDel markers and spanned 1399.40 cM with an average distance of 7.61 cM.

For the three RH-derived populations, leaf samples of each individual were extracted for genomic DNA through the modified CTAB method [31]. To genotype the three RH-derived populations, a total of six InDel markers (Table S2) in the mapping interval of *qGPC1-1* were designed with Primer3.0 (http://primer3.ut.ee/) based on the 30-fold genome resequencing of the parents, HHZ and JZ1560. According to our previous study [32], the PCR was performed in 10-μL reactions containing 2 × *Taq* MasterMix (CW0682, CWBIO) 5-μL, 0.4 μM of each primer and 50 ng DNA template. The PCR program was set as an initial denaturation at 94 °C for 2 min, then followed by 30 cycles of 30 s at 94 °C, 30 s at 55 °C and 30 s at 72 °C, and finally 2 min at 72 °C. The PCR products were analyzed on 2.5% agarose gels.

### 4.4. QTL Mapping and Statistical Analysis

Using the high-density genetic map, QTL analysis for GPC was performed by R/qtl software with the method of composite interval mapping (CIM) [33], taking the two years for the RIL population as two environments. A threshold of LOD > 2.0 was used for detecting a putative QTL. Using the low-density genetic map, QTLs controlling GPC were identified by Windows QTL Cartographer 2.5 [34]. Identification of QTLs was performed using CIM. The LOD threshold for claiming a QTL was also fixed as a LOD score of 2.0. QTL analysis in the three RH-derived populations was also conducted by CIM using Windows QTL Cartographer 2.5 [34].

Basic descriptive statistics, including mean, standard deviation, coefficient of variation, range, skewness and kurtosis, and correlation analysis for GPC in the RIL population were performed with Microsoft Excel 2016 for Windows. The *t*-test of the two parents was performed with the SAS program [35].

## 5. Conclusions

Based on genotyping-by-resequencing, a total of 14 QTLs controlling GPC were identified with an *indica*/*japonica* (HHZ/JZ1560) RIL population in 2016 and 2017. Of the 14 QTLs, 13 QTLs showed similar chromosome regions with the QTLs for GPC documented in previous studies. The *qGPC10* with a minor effect was newly detected in this study. Seven of the fourteen QTLs were repeatedly identified across two years. The stably inherited *qGPC1-1* with a relatively large effect was validated and delimited to a ~862 kb region flanked by JD1006 and JD1075 on the short arm of chromosome 1, which is helpful for the construction of a marker-assisted selection system to improve rice grain qualities and further map-based cloning of this QTL. Our results indicated that instead of the KND method with lengthy operation, the NIRS with rapid and easy operation was a feasible strategy for measuring a massive collection of samples in the mapping and map-based cloning of GPC QTL. More stably expressed QTLs identified in the genetic map based on genotyping-by-resequencing suggested that high-density map enhanced the detection power of minor QTLs.

## Figures and Tables

**Table 1:** Descriptive statistics of the grain protein content (GPC, %) in the recombinant inbred line (RIL) population and the parents in the two years.

Year	Method	RIL Population (*n* = 280)					Parental Mean (*n* = 16)		*p*
		Mean ± SD	CV	Range	Skewness	Kurtosis	HHZ	JZ1560	
2016	NIRS	12.44 ± 1.59	0.13	8.35–16.35	0.20	−0.26	10.14 ± 0.02	13.16 ± 0.01	<0.001
	KND	11.68 ± 1.62	0.14	7.27–15.78	0.18	−0.30	9.39 ± 0.11	13.06 ± 0.33	
2017	NIRS	10.67 ± 1.40	0.13	7.29–15.90	0.28	0.06	8.44 ± 0.03	10.92 ± 0.01	<0.001
	KND	10.64 ± 1.39	0.13	7.39–15.98	0.29	0.15	8.54 ± 0.00	10.62 ± 0.36	

NIRS: near infrared reflectance spectroscopy; KND: Kjeldahl nitrogen determination; *p*: two-tailed *p* value of student’s *t* test.

**Table 2:** Correlation coefficients of GPC between two methods and between different years from 280 RILs.

	16NIRS	16KND	17NIRS
16KND	0.966 **		
17NIRS	0.638 **	0.651 **	
17KND	0.614 **	0.631 **	0.983 **

16NIRS, 17NIRS: GPC of 280 RILs in 2016 and 2017 measured by near infrared reflectance spectroscopy (NIRS); 16KND, 17KND: GPC of 280 RILs in 2016 and 2017 measured by Kjeldahl nitrogen determination (KND). ** highly significant correlations at the 0.01 level.

**Table 3:** QTLs for GPC based on genotyping-by-resequencing in the RIL population.

QTL	Year	Method	Marker Interval	Position (cM)	LOD	*A*	*R*^2^ (%)
*qGPC1-1*	2016	NIRS	Marker37041–37509	36.46	7.96	0.57	9.14
	2016	KND	Marker37041–37509	36.46	9.72	0.55	10.89
	2017	NIRS	Marker37041–37509	36.46	9.59	0.58	11.85
	2017	KND	Marker37508	38.74	8.72	0.47	11.05
*qGPC1-2*	2016	NIRS	Marker138502	205.52	2.12	0.45	5.53
	2016	KND	Marker138502–136654	205.52	2.44	0.38	5.20
	2017	NIRS	Marker136541–138500	205.88	3.07	0.41	5.88
	2017	KND	Marker138502–139278	205.52	3.06	0.36	6.41
*qGPC1-3*	2016	NIRS	Marker208637–210208	255.84	3.00	0.21	1.18
	2016	KND	Marker207139–209159	255.84	2.62	0.15	0.81
*qGPC2*	2016	NIRS	Marker413084–424309	156.64	3.96	−0.36	3.68
	2016	KND	Marker413084–424409	156.64	3.91	−0.31	3.47
*qGPC3-1*	2016	NIRS	Marker449522–449025	5.95	11.15	0.78	16.86
	2016	KND	Marker449522–449025	5.95	12.68	0.72	18.59
	2017	NIRS	Marker450509–451398	7.80	6.34	0.46	7.21
	2017	KND	Marker450216–451025	7.61	6.77	0.41	8.40
*qGPC3-2*	2016	NIRS	Marker567334–573384	91.73	2.41	0.30	2.50
	2016	KND	Marker571649–571070	91.73	2.52	0.26	2.53
	2017	NIRS	Marker571649–572521	91.73	2.31	0.36	4.63
	2017	KND	Marker571649–573384	91.73	2.38	0.30	4.53
*qGPC3-3*	2017	NIRS	Marker620694–621697	133.44	5.74	0.39	5.44
	2017	KND	Marker620694–621697	133.44	6.11	0.36	6.29
*qGPC4*	2016	NIRS	Marker779713-795626	173.67	3.90	0.45	5.61
	2016	KND	Marker790607–796001	178.83	4.58	0.39	5.47
	2017	NIRS	Marker792925–793575	175.36	2.93	0.35	4.26
	2017	KND	Marker792925–796001	175.36	3.56	0.31	4.89
*qGPC5*	2016	NIRS	Marker940773–956686	159.63	4.09	0.35	3.47
	2016	KND	Marker951188–956259	159.63	3.19	0.24	2.12
	2017	NIRS	Marker942375–968984	167.68	5.02	0.41	6.09
	2017	KND	Marker941389–968984	167.68	4.61	0.32	5.21
*qGPC7*	2016	NIRS	Marker1254363–1255283	92.11	3.46	0.50	6.84
	2016	KND	Marker1254363–1255919	92.11	3.83	0.44	7.15
*qGPC8*	2016	NIRS	Marker1330696–1330654	38.51	7.81	−0.57	9.23
	2016	KND	Marker1330714–1331214	39.43	7.10	−0.46	7.71
	2017	NIRS	Marker1330696–1330654	38.51	2.12	−0.28	2.70
	2017	KND	Marker1330696	38.33	2.55	−0.25	3.20
*qGPC10*	2016	NIRS	Marker1714254–1714662	129.12	2.84	−0.43	5.18
*qGPC11-1*	2017	NIRS	Marker1789482–1788848	45.47	2.08	0.33	3.89
*qGPC11-2*	2016	NIRS	Marker1901374–1901256	184.63	2.03	0.46	5.84
	2016	KND	Marker1902918–1902895	186.73	2.06	0.36	4.66

NIRS: near infrared reflectance spectroscopy; KND: Kjeldahl nitrogen determination; *A*: additive effect of replacing a maternal allele with a paternal allele; *R*^2^: proportion of the phenotypic variance explained by the QTL.

**Table 4:** QTLs for the GPC detected in the three residual heterozygote-derived F_2_ populations.

Population Name	Segregating Region	Sample	LOD	*A*	*D*	*R*^2^ (%)
WB01	JD1006–JD1078	180	7.59	0.36	0.11	26.00
WB02	JD1068–JD1007	137	6.25	0.39	0.04	27.40
WB03	JD1075–JD1007	115	ns	ns	ns	ns

*A*: additive effect of replacing a maternal allele with a paternal allele; *D*: dominance effect; *R*^2^: proportion of the phenotypic variance explained by the QTL; ns: no significance.

## Supplementary Materials

Supplementary materials can be found at https://www.mdpi.com/1422-0067/21/2/408/s1.
