# Supplementary material for: Genome-Wide Identification of QTLs for Grain Protein Content Based on Genotyping-by-Resequencing and Verification of qGPC1-1 in Rice
Source: Int J Mol Sci. 2020 Jan 9;21(2):408. doi: 10.3390/ijms21020408 (PMC7014352; doi:10.3390/ijms21020408)
Supplement: Supplementary file 1 [file ijms-21-00408-s001.zip › ijms-662313-supplementary.pdf]

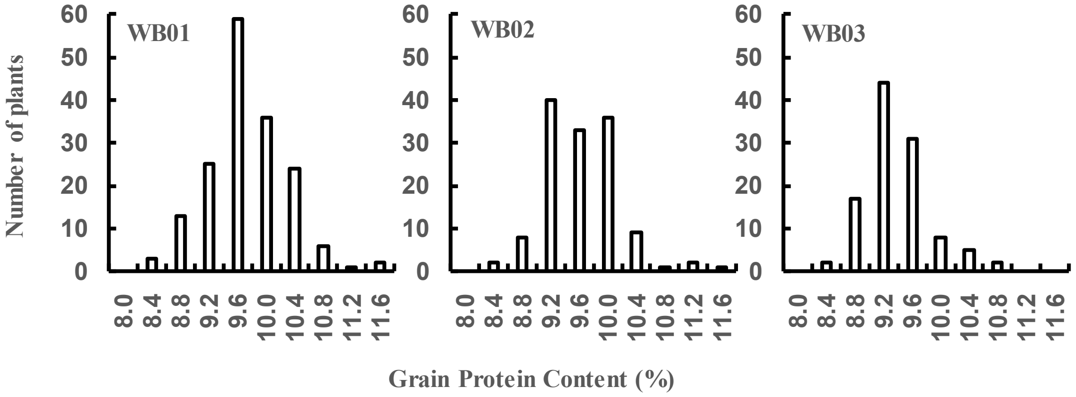

Figure S1. Frequency distributions of grain protein content in three residual heterozygote derived F_2 populations.

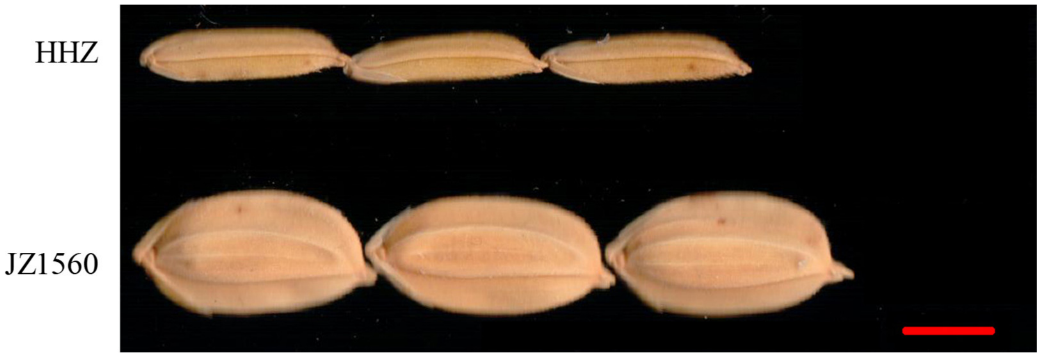

Figure S2. Parental grains. Scale bar, 5 mm. HHZ, huanghuazhan; JZ1560, JiZi1560.

Table S1. QTLs for grain protein content (GPC) based on the low-density genetic map containing 208 SSR and Indel markers in the recombinant inbred line (RIL) population.

QTL	Year	Method	Marker Interval	Position (cM)	LOD	*A*	R^2 (%)
qGPC1	2016	NIRS	JD1006-JD1007	37.11	8.30	0.71	12.40
	2016	KND		38.11	9.06	0.64	13.33
	2017	NIRS		38.11	7.24	0.62	11.78
	2017	KND		38.11	8.07	0.54	12.87
qGPC2-1	2016	NIRS	RM6-JD2008	127.81	2.85	-0.36	3.52
	2016	KND		127.81	2.66	-0.30	3.24
qGPC2-2	2017	NIRS	W004-RM12987	47.61	2.28	0.28	2.90
qGPC3-1	2016	NIRS	JD3002-JD3011	2.91	10.75	0.68	12.74
	2016	KND		2.91	11.37	0.60	12.99
	2017	NIRS		2.91	4.94	0.43	6.06
	2017	KND		2.91	7.33	0.43	9.08
qGPC3-2	2017	NIRS	RM3131-JD3005	35.61	3.45	0.57	6.62
	2017	KND		35.61	3.89	0.49	6.91
qGPC3-3	2016	NIRS	RM2453-RM7370	80.91	2.58	0.34	2.88
qGPC3-4	2017	NIRS	RM2334-RM3525	114.31	5.16	0.53	10.09
	2017	KND		114.31	5.35	0.46	10.49
qGPC4-1	2016	NIRS	RM2636-RM5511	99.71	3.15	0.40	4.51
	2016	KND		102.71	4.12	0.41	6.13
qGPC4-2	2017	NIRS	RM5511-RM1113	112.81	2.49	0.32	3.64
	2017	KND		113.81	2.92	0.30	4.62
qGPC5	2016	NIRS	JD5014-JD5015	95.41	4.02	0.41	4.61
	2016	KND		103.11	2.65	0.30	3.25
	2017	NIRS		103.11	5.87	0.48	8.35
	2017	KND		103.11	4.42	0.35	6.22
qGPC7	2016	NIRS	RM2966-RM3826	53.31	3.26	0.38	3.91
	2016	KND		53.31	2.92	0.31	3.35
qGPC8-1	2016	NIRS	RM1148-RM5556	28.11	6.04	-0.53	7.60
	2016	KND		31.11	5.08	-0.41	5.96
qGPC10	2016	NIRS	RM6745-RM2371	66.21	2.23	-0.32	2.90
qGPC11	2017	NIRS	RM3133-RM5731	25.41	2.13	-0.31	3.32

Position-cM, position of peak marker in cent-Morgan within the interval. A, additive effect of replacing a maternal allele with a paternal allele. R^2(%), proportion of the phenotypic variation explained by the QTL.

Table S2. Sequences and physical location of the markers.

Marker	Physical Position (bp)	Forward Primer	Reverse Primer
JD1037	4995417-4995893	CTCCAATAAAATAGCCCACG	TGCAACAGTTCAACACCAGC
JD1068	6270413-6270652	GGAGACCCGGAGAATTACTT	CATTTCCCTAGACCCTAGCA
JD1038	6515343-6515581	GTTCACGGAATAAGCCAAAC	CCACAGTGCTCATCAAAGAG
JD1075	6835475-6835823	TGTTTCTCTCTTGGCTTTGC	TCCCTTTCCCTTCTCTTGTC
JD1078	7175852-7176049	TCTAAAAGCCTCAACCACTA	CTCTTCTTCATCTCCTCCAT
JD1017	8452950-8453295	TATCCCACCCGATACCTGC	AATAGCCTCCCGCTTCCTT

Physical position of the marker according to the Nipponbare reference database in Rice Release 7.
